# Toxicity of Immune-Checkpoint Inhibitors in Hematological Malignancies

**DOI:** 10.3389/fphar.2021.733890

**Published:** 2021-08-13

**Authors:** Katarina Hradska, Roman Hajek, Tomas Jelinek

**Affiliations:** ^1^ Department of Haematooncology, University Hospital Ostrava, Ostrava, Czechia; ^2^ Faculty of Medicine, University of Ostrava, Ostrava, Czechia

**Keywords:** hematological malignancies, immune checkpoint inhibitors, toxicity, immune related adverse events, PD-1, PD-L1

## Abstract

Immune checkpoint inhibitors (ICIs), especially those targeting the programmed-death 1 (PD-1) receptor and its ligands, have become indispensable agents in solid tumor anti-cancer therapy. Concerning hematological malignancies, only nivolumab and pembrolizumab have been approved for the treatment of relapsed and refractory classical Hodgkin lymphoma and primary mediastinal large B cell lymphoma to date. Nevertheless, clinical research in this field is very active. The mechanism of action of ICIs is based on unblocking the hindered immune system to recognize and eliminate cancer cells, but that also has its costs in the form of ICI-specific immune related adverse events (irAEs), which can affect any organ system and can even be lethal. In this article, we have reviewed all prospective blood cancer clinical trials investigating ICIs (both monotherapy and combination therapy) with available toxicity data with the purpose of determining the incidence of irAEs in this specific setting and to offer a brief insight into their management, as the use of immune checkpoint blockade is not so frequent in hemato-oncology.

## Introduction

In recent years, immunotherapy has become one of the pillars of cancer treatment. Extensive research in cancer biology has led to a deeper understanding of cancer cell survival mechanisms and the importance of the tumor microenvironment. Not only are we gradually uncovering the essential principles of cancerogenesis and immune escape, but we are also using this knowledge therapeutically. One of the most groundbreaking findings is definitely the discovery of tumor cells´ exploitation of immune checkpoints and the introduction of immune checkpoint inhibitors (ICIs).

A normally functioning immune system should be able to distinguish the body´s own antigens from foreign or mutated self-proteins and this executive role is borne by T cells (Buchbinder and Desai, 2016; Jelinek and Hajek, 2016). The vital part of the recognition process is the interaction between a T cell receptor (TCR) and a major histocompatibility complex (MHC) on the surface of the antigen presenting cell (APC), which displays the antigen in question. In addition, there are many other co-stimulatory or co-inhibitory pathways (immune checkpoints) that help to determine the fate of the T lymphocyte. Stimulatory pathways lead to T cells´ activation, maturation, expansion and the inhibition of their apoptosis, so they can eliminate defective or autoreactive cells, whereas inhibitory pathways have the opposite effect, causing T cell anergy and exhaustion and inducing tolerance (Buchbinder and Desai, 2016; Jelinek et al., 2018; Hradska et al., 2020).

The two most important negative regulators of T cell immune function are cytotoxic T lymphocyte-associated antigen 4 (CTLA-4) and programmed death 1 (PD-1) immune checkpoints (Buchbinder and Desai, 2016). CTLA-4 helps to prevent autoreactivity in the early stages of immune response, especially in the lymph nodes, and can be found in the cytoplasm of naïve T cells and on the surface of regulatory T cells (Tregs). Upon binding to its cognate ligands B7-1 and B7-2 on the APCs, it leads to T cell anergy (Buchbinder and Desai, 2016; Matsuki and Younes, 2016; Jelinek et al., 2017; Hradska et al., 2020). PD-1 is typical for “exhausted” T cells that have experienced high levels of stimulation, for example, during chronic infection or cancer, leading to their suboptimal function. However, various immune cells such as B cells and natural killer cells also show PD-1 expression. PD-1 ligands PD-L1 and PD-L2 can be found on various hematopoietic (lymphocytes, APCs) and non-hematopoietic cells (endothelial, parenchymatic cells), but are also induced on many types of tumor cells. PD-1/PD-L1/2 pathway is effective later in the immune response, primarily in peripheral tissues and, under normal circumstances, maintains peripheral tolerance during the course of infection and prevents autoimmunity (Buchbinder and Desai, 2016; Wang et al., 2018).

The rationale behind immune checkpoint blockade is to reinvigorate the immune system, which is inhibited by overexpression of immune checkpoint inhibitor molecules on the surface of cancer cells, to restore T cell cytotoxicity and to destroy defective cells (Jelinek et al., 2018; Hradska et al., 2020). Immune checkpoint inhibitors, monoclonal antibodies (mAbs) targeting PD-1, PD-L1/2 and CTLA-4, were introduced in 2011 and changed the landscape of especially solid tumor cancer treatment so fundamentally that this breakthrough led to the award of the Nobel Prize in 2018 jointly to James P. Allison and Tasuku Honjo (Smyth and Teng, 2018). In hemato-oncology, only two molecules have been approved by the FDA (US Food and Drug Administration) - nivolumab (anti-PD-1 mAb) for the treatment of relapsed/refractory (RR) classical Hodgkin lymphoma (cHL) and pembrolizumab (anti-PD-1 mAb) also for the treatment of RR cHL and for RR primary mediastinal large B cell lymphoma (PMBL) as well (Ansell et al., 2015; Armand et al., 2016, 2018a, 2018b; Chen et al., 2017). However, this is just the tip of the iceberg as plenty of ICIs are investigated in many other hematological malignancies such as non-Hodgkin lymphomas (NHLs), multiple myeloma (MM), acute myeloid leukemia (AML), myelodysplastic syndrome (MDS) and chronic lymphocytic leukemia (CLL).

The new anti-cancer strategy came with a new specific type of toxicity which is known as immune related adverse events (irAEs) (Haanen et al., 2017; Fan et al., 2021). The pathophysiological mechanism is not yet fully understood, but irAEs are considered to be T cell mediated and mark the reinvigorated immune system which may cause organ-specific inflammation, tissue damage and autoimmunity (Chhabra and Kennedy, 2021). Activation of the complement may also play a role in the process (Roth et al., 2021). The most frequently affected tissues are skin, gastrointestinal system, endocrine glands, liver and lungs, but irAEs may involve any organ system. They usually occur within weeks to 3 months after the initiation of ICI therapy and are mild to moderate in severity (Haanen et al., 2017). However, lethal cases are also observed, so it is very important for physicians to recognize potential problems early and to take appropriate measures as soon as possible. IrAEs are classified according to the CTCAE (Common Terminology Criteria for Adverse Events) scoring system.

There are many reviews describing ICIs´ immune related toxicity in solid tumors or in cancer treatment in general. Nevertheless, ICIs are gradually building their position in the treatment of hematological malignancies and, in this article, we would like to overview the incidence of irAEs in this specific setting and also offer a brief insight into the management of irAEs. We took into account all available safety data from blood cancer ICI prospective clinical trials (both monotherapy and combination therapy) until April 2021 using Pubmed database and Google Scholar. The results are divided by affected organ systems.

## Skin Toxicity

Dermatologic toxicity is the most common type of irAE in hematological malignancies. Maculopapular rash, dermatitis and pruritus represent the typical manifestation, but erythema nodosum, vitiligo, xerosis, psoriasis or even exfoliative dermatitis, skin necrosis and Stevens-Johnson syndrome were also observed (Badros et al., 2017; Khouri et al., 2018; Mateos M.-V. et al., 2019, Mateos et al., 2019 M. V.; Diefenbach et al., 2020; Song et al., 2020).

Rash and pruritus occur in 0–17% patients receiving ipilimumab (anti-CTLA-4 mAb) in monotherapy and are mostly mild (grade 1–2). Interestingly, there was no skin toxicity attributed to ipilimumab administered in the post-alogeneic stem cell transplant setting in the study conducted by Davids et al. (2016). Incidence was higher and irAEs slightly more severe in combination therapy, either with another ICI such as nivolumab (18–36%) or with other immunotherapeutic agents (6–61%) (Khouri et al., 2018; Diefenbach et al., 2020). Double immune checkpoint blockade even led to grade 4 Stevens-Johnson syndrome in one patient treated for cHL (Diefenbach et al., 2020).

Nivolumab (anti-PD-1 mAb) was associated with comparable all-grade skin toxicity in monotherapy (9–47%) and in combination with other targeted therapeutics (13–31%) (Armand et al., 2018a; Zinzani et al., 2019; Diefenbach et al., 2020; Maruyama et al., 2020). However, grade 3–4 cases were more common in the second cohort. Lower skin toxicity was observed in 2 combination studies of nivolumab and chemotherapy (AVD - doxorubicin, vinblastine, dacarbazine) for the treatment naïve cHL, where only 0–6% of patients experienced skin irAEs (Ramchandren et al., 2019; Bröckelmann et al., 2020).

Another FDA-approved PD-1 inhibitor, pembrolizumab, caused dermatologic irAEs in 0–33% of patients (Khodadoust et al., 2016; Armand et al., 2019). In the CITN-10 study, it led to worsening of erythema and pruritus in patients with Sezary syndrome, but not in mycosis fungoides (Khodadoust et al., 2016). The flare reaction was associated with high expression of PD-1 on circulating Sézary cells, mostly remitted within 12 weeks with only symptomatic treatment and topical corticosteroids and did not lead to treatment discontinuation. Pembrolizumab combined with immunomodulatory drugs (IMiDs) and dexamethasone was extensively investigated in multiple myeloma. Skin related toxicity was not very frequent (2–10%), but could be considered more severe, because one patient died of treatment-related grade 5 Stevens-Johnson syndrome (D’Souza et al., 2019; Mateos M. V. et al., 2019, Mateos et al., 2019 M.-V.). Interestingly, one myeloma patient developed vitiligo on ICI treatment, which is almost exclusively seen in melanomas (Badros et al., 2017; Ransohoff and Kwong, 2017; Chhabra and Kennedy, 2021).

Anti-PD-L1 mAbs are not yet so widely investigated as anti-PD-1 mAbs, but the skin toxicity seems comparable with rash and dry skin reported from available clinical trial data (Kazandjian et al., 2021; Ribrag et al., 2021). (Table 1)

**TABLE 1:** Incidence of skin irAEs in hematological malignancies.

Skin Toxicity						
Author	Condition	Phase	Regimen	Symptom	Incidence	
					all grade	grade ≥ 3
Armand et al. (2021)	RR cHL, NHL, MM	1b	nivo + ipili	skin toxicity	28%	NA
			nivo + liri		24%	NA
Diefenbach et al. (2020)	RR cHL	1/2	ipili + BV	pruritus/rash	30%/61%	4%/22%
			nivo + BV		26%/31%	5%/5%
			ipili + nivo + BV		18%/36%	5%/9%
			ipili + nivo + BV	Stevens-Johnson sy	5%	5%
Tuscano et al. (2019)	RR B-NHL	1	ipili + R	rash	24%	3%
Khouri et al. (2018)	RR lymphoid malign.	2	ipili + lena	dermatitis	6%	0%
Ansell et al. (2009)	RR B-NHL	1	ipili	pruritus/rash	11%/17%	0%/0%
Ribrag et al. (2021)	RR DLBCL	1b	durva + tremeli	dry skin	33%	NA
Maruyama et al. (2020)	RR cHL	2	nivo	pruritus/rash	35%/47%	0%/6%
Zinzani et al. (2019)	RR PMBL	1/2	nivo + BV	rash	13%	3%
Ramchandren et al. (2019)	ND cHL	2	nivo + AVD	rash	6%	0%
Younes et al. (2019)	RR NHL, RR CLL	1/2a	nivo + ibru	rash	NA	8%
Ansell et al. (2019)	RR DLBCL	2	nivo	rash	NA	2%
Armand et al. (2018a)	RR cHL	2	nivo	rash	9%	2%
Herrera et al. (2018)	RR cHL	1/2	nivo + BV	rash	NA	2%
Lesokhin et al. (2016)	RR hematol. malign.	1b	nivo	skin tox	18%	1%
Ansell et al. (2015)	RR cHL	1	nivo	pruritus/rash	13%/22%	0%/0%
Khodadoust et al. (2016)	RR MF, RR SS	2	pembro	rash + skin flare	42%	17%
Barta et al. (2019)	RR T-NHL	2	pembro	rash	17%	11%
Armand et al. (2016)	RR cHL	1b	pembro	xerosis	2%	0%
Liu et al. (2021)	RR cHL	2	camre + decitabine	rash	12%	0%
Mei et al. (2020)	RR PMBL	2	camre + GVD	pruritus/rash	30%/22%	0%/0%
Kazandjian et al. (2021)	RR MM	2	avelumab	rash	25%	0%
D’Souza et al. (2019)	ND MM	2	pembro + lena	rash	10%	0%
Usmani et al. (2019)	ND MM	3	pembro + lena + dex	rash	9%	9%
Mateos et al. (2019a)	RR MM	1	pembro + lena + dex	exfoliative dermatitis	2%	0%
Ribrag et al. (2019)	RR MM	1b	pembro	pruritus	3%	0%
Mateos et al. (2019b)	RR MM	3	pembro + pom + dex	rash	3%	3%
				Stevens-Johnson sy	1%	1%
Badros et al. (2017)	RR MM	2	pembro + pom + dex	vitiligo	2%	0%
Zeidan et al. (2018)	RR MDS	1b	ipili	dermatitis + rash	14%	3%
Davids et al. (2020)	RR hematol. malign.	1	nivo	rash	29%	4%
Ravandi et al. (2019)	ND AML, ND MDS	2	nivo + ida + cytarabine	rash	NA	5%
Cassaday et al. (2020)	ND/RR ALL	2	pembro	Stevens-Johnson sy	8%	8%

RR, relapsed/refractory; ND, newly diagnosed; cHL, classical Hodgkin lymphoma; NHL, non-Hodgkin lymphoma; MM, multiple myeloma; PMBL, primary mediastinal large B cell lymphoma; CLL, chronic lymphocytic leukemia; DLBCL, diffuse large B cell lymphoma; FL, follicular lymphoma; MF, mycosis fungoides; SS, Sézary syndrome; AML, acute myeloid leukemia; MDS, myelodysplastic syndrome; ALL, acute lymphoblastic leukemia; ipili, ipilimumab; nivo, nivolumab; BV, brentuximab vedotin; liri, lirilumab; R, rituximab; AVD, adriamycin + vinblastine + dacarbazine; GVD, gemcitabine + vinorelbine + doxorubicin; camre, camrelizumab; pembro, pembrolizumab; ibru, ibrutinib; lena, lenalidomide; pom, pomalidomide; dex, dexamethasone; ida, idarubicin; durva, durvalumab; tremeli, tremelimumab; NA, not available.

Grade 1 (affecting <10% of body surface area = BSA) and grade 2 (10–30% BSA) dermatologic irAEs are typically managed with topical emollients, topical steroids and/or antihistamines, avoiding skin irritants or sun exposure and usually do not lead to ICI treatment discontinuation (Haanen et al., 2017; Chhabra and Kennedy, 2021). More severe cases are associated with affection of >30% BSA and cooperation with a dermatology specialist and punch biopsy is recommended. Grade 3 cases are managed with oral (prednisolone 0.5–1 mg/kg) or intravenous (methylprednisolone 0.5–1 mg/kg) systemic steroids and, after resolving the symptoms to grade 1 or mild grade 2, ICIs can be restarted after thorough risk/benefit assessment and discussion with the patient. Steroids are recommended to be weaned over 1–2 and 2–4 weeks respectively, because of the risk of relapse. Grade 4 is a life-threatening situation associated with symptoms such as erythema, purpura or epidermal detachment, requires rapid administration of intravenous steroids (methylprednisolone 1–2 mg/kg) and permanent discontinuation of ICI treatment.

## Endocrine Toxicity

Patients receiving ICI treatment relatively often experience irAEs affecting endocrine organs - mostly the thyroid gland, pituitary gland, adrenal glands and pancreas (Chang et al., 2019; Chhabra and Kennedy, 2021). Anti-CTLA-4 mAbs are connected with higher rates of hypophysitis, whereas PD-1 inhibitors more frequently induce hypothyroidism, hyperthyroidism or thyroiditis. Type 1 diabetes mellitus and adrenal insufficiency are less common.

Ipilimumab use in hematological malignancies, both in monotherapy or combination therapy, was associated with low incidence of thyroid dysfunction (0–6%) (Davids et al., 2016; Khouri et al., 2018). Only one case of suspected mild hypophysitis was reported in a phase 1 study for RR B-NHL (Ansell et al., 2009). This patient with a history of type 2 diabetes mellitus developed hyperglycemia during upper respiratory tract infection, anorexia, anemia, weight loss and erectile dysfunction. Laboratory findings uncovered low serum testosterone, low-normal gonadotropin levels and a normal MRI pituitary scan.

Both FDA-approved PD-1 inhibitors, nivolumab and pembrolizumab, showed similar thyroid toxicity. Hypothyroidism occurred in 0–29% and 0–17% of cases, respectively, while hyperthyroidism was reported in 0–13% and 0–17% of cases, respectively (Khodadoust et al., 2016; D’Souza et al., 2019; Younes et al., 2019; Zinzani et al., 2019; Maruyama et al., 2020). Adrenal insufficiency was also observed in both of these agents with the maximum incidence of 6% (Badros et al., 2017; Armand et al., 2018a; Barta et al., 2019; Ribrag et al., 2019). Type 1 diabetes mellitus was associated with nivolumab use in two RR cHL clinical trials (1 patient each) (Armand et al., 2018a; Maruyama et al., 2020). The rare endocrine irAE, hypoparathyroidism, affected one patient with cHL while receiving camrelizumab (anti-PD-1 mAb) and decitabine (hypomethylating agent) combination treatment (Liu et al., 2021). There was no endocrine toxicity observed in anti-PD-L1 based hematological clinical trials and in all available studies investigating ICI use in acute leukemia or myelodysplastic syndrome (Ravandi et al., 2019; Cassaday et al., 2020; Kazandjian et al., 2021; Ribrag et al., 2021). (Table 2)

**TABLE 2:** Incidence of endocrine irAEs in hematological malignancies.

Endocrine Toxicity						
Author	Condition	Phase	Regimen	Symptom	Incidence	
					all grade	grade ≥ 3
Diefenbach et al. (2020)	RR cHL	1/2	ipili + nivo + BV	endocrine disorders	5%	5%
Khouri et al. (2018)	RR lymphoid malign.	2	ipili + lena	hypothyroidism	6%	0%
				hyperthyroidism	6%	0%
Bashey et al. (2009)	cancer post HSCT	1	ipili	hyperthyroidism	3%	0%
Ansell et al. (2009)	RR B-NHL	1	ipili	hypophysitis susp.	8%	0%
Maruyama et al. (2020)	RR cHL	2	nivo	hypothyroidism	29%	0%
				fulminant DM 1T	6%	6%
Bröckelmann et al. (2020)	ND cHL	2	nivo + AVD	hypothyroidism	7%	0%
Zinzani et al. (2019)	RR PMBL	1/2	nivo + BV	hyperthyroidism	13%	0%
				hypothyroidism	7%	0%
				thyroiditis	3%	0%
Ramchandren et al. (2019)	ND cHL	2	nivo + AVD	hyperthyroidism	8%	0%
				hypothyroidism/thyroiditis	18%	0%
Armand et al. (2018a)	RR cHL	2	nivo	hyperthyroidism	2%	0%
				hypothyroidism/thyroiditis	12%	0%
				DM	1%	1%
				adrenal insufficiency	1%	0%
Ansell et al. (2015)	RR cHL	1	nivo	hypothyroidism	9%	0%
Armand et al. (2019)	RR PMBL	1b	pembro	hypothyroidism	9%	0%
		2		hypothyroidism	8%	0%
				hyperthyroidism	4%	0%
				thyroiditis	2%	0%
Barta et al. (2019)	RR T-NHL	2	pembro	hypothyroidism	11%	11%
				adrenal insufficiency	6%	NA
Armand et al. (2016)	RR cHL	1b	pembro	hypothyroidism	16%	0%
				thyroiditis	6%	0%
Liu et al. (2021)	RR cHL	2	camre	hypothyroidism	16%	0%
			camre + decitabine	hypothyroidism	7%	0%
				hypoparathyroidism	2%	0%
D’Souza et al. (2019)	ND MM	2	pembro + lena	hypothyroidism	17%	0%
				hyperthyroidism	17%	0%
Usmani et al. (2019)	ND MM	3	pembro + lena + dex	hypothyroidism	7%	0%
				hyperthyroidism	6%	2%
Mateos et al. (2019a)	RR MM	1	pembro + lena + dex	hypothyroidism	8%	0%
				hyperthyroidism	6%	0%
Ribrag et al. (2019)	RR MM	1b	pembro	hypothyroidism	3%	0%
Mateos et al. (2019b)	RR MM	3	pembro + pom + dex	hyperthyroidism	3%	0%
				hypothyroidism	2%	0%
Badros et al. (2017)	RR MM	2	pembro + pom + dex	hypothyroidism	10%	4%
				adrenal insufficiency	4%	2%

RR, relapsed/refractory; ND, newly diagnosed; cHL, classical Hodgkin lymphoma; NHL, non-Hodgkin lymphoma; MM, multiple myeloma; PMBL, primary mediastinal large B cell lymphoma; CLL, chronic lymphocytic leukemia; DLBCL, diffuse large B cell lymphoma; FL, follicular lymphoma; HSCT, (allogeneic) hematopoietic stem cell transplantation; ipili, ipilimumab; nivo, nivolumab; BV, brentuximab vedotin; AVD, adriamycin + vinblastine + dacarbazine; camre, camrelizumab; pembro; pembrolizumab; lena, lenalidomide; pom, pomalidomide; dex, dexamethasone; susp., suspect; DM, diabetes mellitus; DM 1T, type 1 diabetes mellitus; NA, not available.

Endocrine disorders during ICI therapy are usually mild to moderate (grade 1 and 2), but in a minority of cases, can be severe or life-threatening (Haanen et al., 2017; Chang et al., 2019; Chhabra and Kennedy, 2021). Thyroid-targeting irAEs are seen in up to 80% of cases caused by anti-thyroid antibodies. Hyperthyroidism is often transient and within 3–6 weeks usually leads to permanent hypothyroidism which does not resolve after ICI discontinuation and requires life-long thyroid hormone replacement. It is recommended to perform thyroid function tests (TSH = thyroid-stimulating hormone, fT4 = free thyroxine, fT3 = free tri-iodothyronine) every cycle for the first 3 months of anti-PD-(L)1 treatment and every second cycle thereafter. Cortisol level should be taken when TSH is falling and/or symptoms suggesting secondary (central) hypothyroidism occur. It can be a sign of hypopituitarism and potentially life-threatening central adrenal insufficiency when rapid corticosteroid replacement prevents development of adrenal crisis. In the rare cases of symptomatic thyreotoxicosis, beta-blockers (propranolol or atenolol) are recommended. Antithyroid drugs are not routinely used unless Graves´ disease is diagnosed. In the case of painful thyroiditis, prednisolone in the dosage of 0.5 mg/kg should be considered. Thyroid disorders usually do not lead to ICI discontinuation.

Blood glucose levels should be monitored regularly in patients treated with ICIs because of the danger of *de novo* type 1 diabetes mellitus (Haanen et al., 2017; Chang et al., 2019). Patients with pre-existing type 2 diabetes mellitus can develop diabetic ketoacidosis. These complications should be managed according to local practice; high-dose steroids are not recommended. Affected patients can restart ICI treatment after they are stable and insulin therapy is adjusted. ICI discontinuation does not lead to the restitution of the endogenous insulin level.

Primary adrenal insufficiency (PAI) differs from abovementioned central adrenal insufficiency by elevated ACTH (adrenocorticotropic hormone) and low cortisol level (Chang et al., 2019). The difference is also in management, as PAI requires rapid mineralocorticoid replacement in addition to glucocorticoids.

## Hepatotoxicity

ICI related liver injury shares many features with autoimmune hepatitis. In most cases, it presents with asymptomatic elevation of alanine aminotransferase (ALT), aspartate aminotransferase (AST) or total bilirubin, but severe hepatitis and liver failure associated with fever, malaise, jaundice and change of stool color can also occur (Tian et al., 2018; Chhabra and Kennedy, 2021).

The incidence of abnormal ALT and AST levels is comparable between CTLA-4 and PD-1 inhibitors. The most prominent treatment-related hepatotoxicity was observed in a phase 1/2 clinical trial investigating the efficacy of ipilimumab, nivolumab and brentuximab vedotin (BV) in RR cHL patients - the rate of ALT/AST increase was as high as 48%/39% for the ipilimumab + BV cohort and 47%/32% for the nivolumab + BV cohort (Diefenbach et al., 2020). All of these events were grade 1 to 2 in severity. Singular grade 3 cases were detected in the triple therapy group. Slightly less toxic anti-PD-1 mAbs appeared to be pembrolizumab and camrelizumab with only 0–8% and 0% proportion of patients experiencing liver dysfunction, respectively (Khodadoust et al., 2016; Armand et al., 2019; Mei et al., 2020; Liu et al., 2021). Hepatotoxicity was also reported in anti-PD-L1 hemato-oncological trials, but the cohorts were small, so the results should be regarded with caution (Kazandjian et al., 2021; Ribrag et al., 2021). (Table 3)

**TABLE 3:** Incidence of hepatic irAEs in hematological malignancies.

Hepatotoxicity						
Author	Condition	Phase	Regimen	Symptom	Incidence	
					all grade	grade ≥ 3
Armand et al. (2021)	RR cHL, NHL, MM	1b	nivo + ipili	elevated ALT/AST	NA	3%/2%
Diefenbach et al. (2020)	RR cHL	1/2	ipili + BV	elevated ALT/AST	48%/39%	0%/0%
			nivo + BV		47%/32%	0%/0%
			ipili + nivo + BV		14%/14%	5%/5%
Ansell et al. (2009)	RR B-NHL	1	ipili	elevated AST	22%	0%
Maruyama et al. (2020)	RR cHL	2	nivo	abnormal hepatic function	12%	6%
Zinzani et al. (2019)	RR PMBL	1/2	nivo + BV	hepatitis	3%	3%
Ramchandren et al. (2019)	ND cHL	2	nivo + AVD	hepatitis	4%	4%
				elevated ALT/AST	4%/2%	4%/2%
Younes et al. (2019)	RR NHL, RR CLL	1/2a	nivo + ibru	elevated ALT/AST	NA/NA	2%/1%
Ansell et al. (2019)	RR DLBCL	2	nivo	abnormal hepatic function	NA	3%
Armand et al. (2018a)	RR cHL	2	nivo	hepatitis	5%	4%
Herrera et al., 2018	RR cHL	1/2	nivo + BV	elevated ALT/AST	NA/NA	2%/2%
Lesokhin et al. (2016)	RR hematol. malign.	1b	nivo	elevated ALT + AST	2%	0%
Khodadoust et al. (2016)	RR MF, RR SS	2	pembro	elevated ALT/AST	4%/8%	4%/4%
Barta et al. (2019)	RR T-NHL	2	pembro	abnormal hepatic function	6%	0%
Armand et al. (2016)	RR cHL	1b	pembro	elevated ALT/AST	6%/6%	3%/3%
Ribrag et al. (2021)	RR DLBCL	1b	durva + danvatirsen	elevated ALT/AST	33%/29%	NA/NA
Kazandjian et al. (2021)	RR MM	2	avelumab	elevated ALT/AST	25%/25%	0%/0%
D’Souza et al. (2019)	ND MM	2	pembro + lena	hepatitis	3%	3%
Usmani et al. (2019)	ND MM	3	pembro + lena + dex	hepatitis	1%	1%
				drug-induced liver injury	1%	1%
Mateos et al. (2019a)	RR MM	3	pembro + pom + dex	hepatitis	1%	1%
Badros et al. (2017)	RR MM	2	pembro + pom + dex	hepatitis	4%	2%
Zeidan et al. (2018)	RR MDS	1b	ipili	transaminitis	7%	7%
Davids et al. (2020)	RR hematol. malign.	1	nivo	transaminitis	29%	7%
Ravandi et al. (2019)	ND AML, ND MDS	2	nivo + ida + cytarabine	transaminitis	NA	3%
Daver et al. (2019)	RR AML	2	nivo + azacitidine	transaminitis	NA	3%

RR, relapsed/refractory; ND, newly diagnosed; cHL, classical Hodgkin lymphoma; NHL, non-Hodgkin lymphoma; MM, multiple myeloma; PMBL, primary mediastinal large B cell lymphoma; CLL, chronic lymphocytic leukemia; DLBCL, diffuse large B cell lymphoma; FL, follicular lymphoma; MF, mycosis fungoides; SS, Sézary syndrome; AML, acute myeloid leukemia; MDS, myelodysplastic syndrome; ALL, acute lymphoblastic leukemia; ipili, ipilimumab; nivo, nivolumab; BV, brentuximab vedotin; AVD, adriamycin + vinblastine + dacarbazine; pembro, pembrolizumab; lena, lenalidomide; pom, pomalidomide; dex, dexamethasone; ibru, ibrutinib; ida, idarubucin; durva, durvalumab; ALT, alanine aminotransferase; AST, aspartate aminotransferase ; NA, not available.

When considering hepatic irAE, other causes of liver injury such as medication, autoimmunity, viral infection and alcohol should be ruled out in the first place (Haanen et al., 2017; Tian et al., 2018; Chhabra and Kennedy, 2021). Imaging methods (CT = computer tomography, MRI = magnetic resonance imaging, Doppler ultrasound) or liver biopsy could help in disputable situations. Management is dependent on severity of damage. Grade 1 (1-3x ULN = upper limit of normal) cases are closely monitored at least once weekly and ICI treatment can continue. When ALT/AST levels reach grade 2 (3-5x ULN), ICI should be temporary withheld and oral corticosteroid therapy initiated (prednisolone 1 mg/kg). Grade 3 and 4 liver injuries (5-20x ULN and >20 ULN, respectively) lead to permanent ICI discontinuation and systemic steroid treatment. In grade 3 cases with ALT/AST<400, normal bilirubin, INR (International Normalized Ratio) and albumin oral prednisolone 1 mg/kg is suggested. Higher dosage (2 mg/kg) of prednisolone should be administered when there is ALT/AST>400, elevated bilirubin or INR or decreased albumin. Grade 4 toxicity is managed by (methyl)prednisolone 2 mg/kg. Steroid-refractory patients might benefit from mycophenolate mofetil (500–1000 mg twice a day), tacrolimus or anti-thymocyte globulin. Infliximab is not recommended due to its potential hepatotoxic effect.

## Gastrointestinal Toxicity

Gastrointestinal (GI) irAEs occur frequently, especially during anti-CTLA-4 mAb treatment (Rocha et al., 2019; Chhabra and Kennedy, 2021). Their clinical presentation can be variable with diarrhoea, abdominal pain, hematochezia, weight loss, fever, vomiting, mouth ulcers, anal lesions and even extra-intestinal manifestations reported (Haanen et al., 2017). Upper GI symptoms (nausea, vomiting, dysphagia, epigastric pain) are less common (Haanen et al., 2017; Rocha et al., 2019).

In hemato-oncological clinical trials, ipilimumab showed similar GI toxicity as in solid tumors. Diarrhoea was the most usual manifestation, observed in a maximum of 56 and 61% patients receiving ipilimumab monotherapy or combination therapy, respectively (Ansell et al., 2009; Diefenbach et al., 2020). Colitis was reported in a maximum of 7 and 5%, respectively (Zeidan et al., 2018; Diefenbach et al., 2020). Double immune checkpoint blockade (ipilimumab + nivolumab) was associated also with gastritis and a severe case of autoimmune pancreatitis (Diefenbach et al., 2020; Armand et al., 2021).

Anti-PD-1 and anti-PD-L1 agents seemed to be less toxic to the GI tract (Chhabra and Kennedy, 2021). Diarrhoea was also the most commonly reported GI symptom, but the incidence did not exceed 33% (Ribrag et al., 2021). Other types of manifestation included colitis, typhlitis, enteritis, proctitis, proctosigmoiditis, pancreatitis, cholecystitis and duodenitis (Khodadoust et al., 2016; Armand et al., 2019; Ravandi et al., 2019; Diefenbach et al., 2020; Maruyama et al., 2020). No grade 5 GI ICI-related toxicity has been reported so far. (Table 4)

**TABLE 4:** Incidence of gastrointestinal irAEs in hematological malignancies.

Gastrointestinal Toxicity						
Author	Condition	Phase	Regimen	Symptom	Incidence	
					all grade	grade ≥ 3
Armand et al. (2021)	RR cHL, NHL, MM	1b	nivo + ipili	diarrhoea	19%	2%
				colitis	2%	2%
				AI pancreatitis	2%	2%
			nivo + liri	diarrhoea	11%	0%
Diefenbach et al. (2020)	RR cHL	1/2	ipili + BV	diarrhoea	61%	4%
			nivo + BV	diarrhoea	21%	0%
				typhlitis	5%	5%
			ipili + nivo + BV	diarrhoea	45%	5%
				colitis	5%	5%
				gastritis	10%	5%
Tuscano et al. (2019)	RR B-NHL	1	ipili + R	diarrhoea	30%	12%
				colitis	3%	3%
Davids et al. (2016)	RR hematol. malign.	1/1b	ipili	diarrhoea	4%	0%
				colitis	4%	4%
Ansell et al. (2009)	RR B-NHL	1	ipili	diarrhoea	56%	28%
Maruyama et al. (2020)	RR cHL	2	nivo	diarrhoea	6%	0%
				enteritis/proctitis	6%/12%	0%/6%
Bröckelmann et al. (2020)	ND cHL	2	nivo + AVD	proctosigmoiditis	1%	1%
Ramchandren et al. (2019)	ND cHL	2	nivo + AVD	diarrhoea	10%	0%
Zinzani et al. (2019)	RR PMBL	1/2	nivo + BV	colitis	3%	3%
Younes et al. (2019)	RR NHL, RR CLL	1/2a	nivo + ibru	diarrhoea	NA	1%
				colitis/enterocolitis	NA	1%/1%
Ansell et al. (2019)	RR DLBCL	2	nivo	diarrhoea	NA	3%
Armand et al. (2018a)	RR cHL	2	nivo	diarrhoea	2%	1%
				colitis	2%	1%
Herrera et al., 2018	RR cHL	1/2	nivo + BV	diarrhoea	NA	3%
				colitis	NA	3%
Lesokhin et al. (2016)	RR hematol. malign.	1b	nivo	diarrhoea + enteritis	7%	0%
Ansell et al. (2015)	RR cHL	1	nivo	diarrhoea	13%	0%
				pancreatitis	4%	4%
Khodadoust et al. (2016)	RR MF, RR SS	2	pembro	colitis + duodenitis	8%	4%
Barta et al. (2019)	RR T-NHL	2	pembro	diarrhoea	6%	0%
Armand et al. (2019)	RR PMBL	1b	pembro	colitis	5%	0%
Armand et al. (2016)	RR cHL	1b	pembro	diarrhoea/colitis	16%/NA	NA/3%
Liu et al. (2021)	RR cHL	2	camre + decitabine	diarrhoea	10%	0%
Ribrag et al. (2021)	RR DLBCL	1b	durva + tremeli	diarrhoea	33%	NA
D’Souza et al. (2019)	ND MM	2	pembro + lena	colitis	13%	3%
Usmani et al. (2019)	ND MM	3	pembro + lena + dex	colitis/pancreatitis	2%/1%	1%/1%
Zeidan et al. (2018)	RR MDS	1b	ipili	colitis	7%	3%
Davids et al. (2020)	RR hematol. malign.	1	nivo	diarrhoea	11%	0%
Ravandi et al. (2019)	ND AML, ND MDS	2	nivo + ida + cytarabine	colitis/pancreatitis	NA/NA	5%/3%
				cholecystitis	NA	3%
Daver et al. (2019)	RR AML	2	nivo + azacitidine	colitis/enterocolitis	1%/1%	1%/0%

RR, relapsed/refractory; ND, newly diagnosed; cHL, classical Hodgkin lymphoma; NHL, non-Hodgkin lymphoma; MM, multiple myeloma; PMBL, primary mediastinal large B cell lymphoma; CLL, chronic lymphocytic leukemia; DLBCL, diffuse large B cell lymphoma; MF, mycosis fungoides; SS, Sézary syndrome; AML, acute myeloid leukemia; MDS, myelodysplastic syndrome; ipili, ipilimumab; liri, lirilumab; nivo, nivolumab; BV, brentuximab vedotin; AVD, adriamycin + vinblastine + dacarbazine; R, rituximab; pembro, pembrolizumab; lena, lenalidomide; dex, dexamethasone; ibru, ibrutinib; ida, idarubucin; durva, durvalumab; tremeli, tremelimumab; camre, camrelizumab; AI, autoimmune; NA, not available.

When considering GI toxicity of ICIs, bacterial or viral infection should be excluded at first (Haanen et al., 2017; Rocha et al., 2019; Chhabra and Kennedy, 2021). GI involvement associated with the main diagnosis is not as frequently seen in hematological malignancies as in solid oncology, when metastases could cause symptoms similar to GI irAEs. Laboratory tests, abdominal X ray or CT scan and endoscopic methods could help in dubious cases. The benefit of biopsy should be carefully assessed due to the risk of perforation. GI irAEs may mimic idiopathic inflammatory bowel disease.

Grade 1 colitis is defined by < 4 liquid stools per day and the treatment is symptomatic with mainly fluid and electrolyte repletion (Haanen et al., 2017; Chhabra and Kennedy, 2021). When grade 1 colitis persists >14 days or grade 2 (4–6 liquid stools) persists >3 days, ICI treatment should be withheld and oral steroids (prednisolone 0.5–1 mg/kg or budesonide 9mg once daily) immediately initiated. If no improvement is seen after 72 h or in grade 3–4 cases (≥7 liquid stools per day), intravenous (methyl)prednisolone 1–2 mg/kg is recommended with possible escalation to infliximab 5 mg/kg after another 72 h. Other possible immunosuppressive treatment options include mycophenolate mofetil (500–1000 mg twice a day) or tacrolimus. Patients experiencing grade 3–4 GI toxicity are not usually re-challenged with ICI therapy because of the high risk of relapse, but it could be discussed on an individual basis.

## Pneumotoxicity

Pulmonary toxicity of ICIs does not occur very often, but can be rapidly progressive and even fatal when present (Chhabra and Kennedy, 2021). Generally, it is more typical for anti-PD-(L)1 mAbs in comparison to anti-CTLA-4 inhibitors. Pneumonitis is one of the most common reasons for ICI discontinuation and in hematological malignancies, it is the leading treatment-related cause of death. Clinical symptoms include dyspnea, cough and chest pain and the median time to onset is 10–12 weeks after the initiation of ICI treatment.

The incidence of pneumotoxicity for ipilimumab monotherapy was 0–11%, and 5–12% for the combination with nivolumab (Ansell et al., 2009; Davids et al., 2016; Diefenbach et al., 2020; Armand et al., 2021). There was one grade 5 case in each group (Davids et al., 2016; Diefenbach et al., 2020). Nivolumab and pembrolizumab caused pneumonitis/interstitial lung disease/upper respiratory tract infection in 0–24% and 0–13% of patients, respectively (Ansell et al., 2015; Badros et al., 2017; Armand et al., 2019; Maruyama et al., 2020). There were 4 treatment-related deaths reported for the nivolumab cohort, while none for pembrolizumab (Lesokhin et al., 2016; Daver et al., 2019; Ramchandren et al., 2019; Diefenbach et al., 2020). No pneumotoxicity was observed in either of the anti-PD-L1 mAb clinical trials (avelumab, durvalumab + tremelimumab) (Kazandjian et al., 2021; Ribrag et al., 2021). (Table 5).

**TABLE 5:** Incidence of pulmonary irAEs in hematological malignancies.

Pneumotoxicity						
Author	Condition	Phase	Regimen	Symptom	Incidence	
					all grade	grade ≥ 3
Armand et al. (2021)	RR cHL, NHL, MM	1b	nivo + ipili	pneumonitis	12%	5%
			nivo + liri		1%	0%
Diefenbach et al. (2020)	RR cHL	1/2	ipili + BV	pneumonitis	4%	0%
			nivo + BV		10%	10%
			ipili + nivo + BV		5%	0%
Davids et al. (2016)	RR hematol. malign.	1/1b	ipili	pneumonitis	11%	4%
Bashey et al. (2009)	cancer post HSCT	1	ipili	pneumonitis	7%	7%
Maruyama et al. (2020)	RR cHL	2	nivo	IST lung disease	24%	6%
				pneumonitis	12%	0%
Bröckelmann et al. (2020)	ND cHL	2	nivo + AVD	pneumonitis	1%	0%
Armand et al. (2018a)	RR cHL	2	nivo	pneumonitis	4%	0%
Herrera et al., 2018	RR cHL	1/2	nivo + BV	pneumonitis	NA	3%
Lesokhin et al. (2016)	RR hematol. malign.	1b	nivo	pneumonitis	11%	4%
Khodadoust et al. (2016)	RR MF, RR SS	2	pembro	pneumonitis	8%	4%
Barta et al. (2019)	RR T-NHL	2	pembro	pneumonitis	11%	11%
Armand et al. (2019)	RR PMBL	2	pembro	pneumonitis	2%	2%
Armand et al. (2016)	RR cHL	1b	pembro	pneumonitis	10%	0%
Liu et al. (2021)	RR cHL	2	camre	pneumonitis	5%	5%
			camre + decitabine		5%	0%
Mei et al. (2020)	RR PMBL	2	camre + GVD	pneumonitis	7%	4%
Usmani et al. (2019)	ND MM	3	pembro + lena + dex	pneumonitis	1%	0%
Ribrag et al. (2019)	RR MM	1b	pembro	upper respir. tract inf.	3%	0%
Mateos et al. (2019a)	RR MM	3	pembro + pom + dex	pneumonitis	3%	1%
Badros et al. (2017)	RR MM	2	pembro + pom + dex	pneumonitis	13%	2%
Zeidan et al. (2018)	RR MDS	1b	ipili	pneumonitis	3%	0%
Davids et al. (2020)	RR hematol. malign.	1	nivo	pneumonitis	14%	4%
Daver et al. (2019)	RR AML	2	nivo + azacitidine	pneumonitis	13%	1%

RR, relapsed/refractory; ND, newly diagnosed; cHL, classical Hodgkin lymphoma; NHL, non-Hodgkin lymphoma; MM, multiple myeloma; PMBL, primary mediastinal large B cell lymphoma; DLBCL, diffuse large B cell lymphoma; MF, mycosis fungoides; SS, Sézary syndrome; AML, acute myeloid leukemia; MDS, myelodysplastic syndrome; HSCT, (allogeneic) hematopoietic stem cell transplantation; ipili, ipilimumab; liri, lirilumab; nivo, nivolumab; BV, brentuximab vedotin; AVD, adriamycin + vinblastine + dacarbazine; GVD, gemcitabine + vinorelbine + doxorubicin; pembro, pembrolizumab; pom, pomalidomide; dex, dexamethasone; ida, idarubucin; camre, camrelizumab; IST, interstitial.

Pneumonitis is a diagnosis per exclusionem, so other conditions such as pulmonary embolism, pneumonia, viral infection or cancer progression should be excluded in the first place (Chhabra and Kennedy, 2021). The diagnostic method of choice is a high-resolution CT scan where ground glass opacities, organizing pneumonia, interstitial pneumonia or hypersensitivity pneumonitis could support the diagnosis of ICI-related lung toxicity (Haanen et al., 2017; Chhabra and Kennedy, 2021). Bronchoscopy with bronchoalveolar lavage may help to rule out infection. Lung biopsy is recommended only in dubious cases and it should be performed by video-assisted thoracoscopic surgery (Haanen et al., 2017; Delaunay et al., 2019).

Grade 1 pneumonitis is characterized by radiological findings without clinical symptoms (Haanen et al., 2017; Delaunay et al., 2019; Chhabra and Kennedy, 2021). These patients should be closely monitored and the delay of treatment considered. Oral prednisone 1 mg/kg and ICI interruption are recommended when the symptoms arise (grade 2). After recovery, steroids should be tapered over a period of 4–6 weeks due to possible relapse. Patients can be re-challenged with ICI when the dose of oral prednisone is ≤ 10 mg. Grade 3 and 4 cases of pneumonitis are associated with new or worsening hypoxia and life-threatening breathing difficulties. These patients should be hospitalized and high-dose intravenous (methyl)prednisolone (2–4 mg/kg/day) initiated. If there is no improvement after 2 days, infliximab (5 mg/kg), mycophenolate mofetil (1000 mg twice a day) or cyclophosphamide are to be added. Slow steroid tapering over at least 6 weeks is also recommended upon recovery to avoid relapse. In grade ≥3 cases when the infectious etiology can´t be quickly dismissed, broad-spectrum antibiotics are to be initiated alongside steroids. Restarting ICI therapy in patients experiencing severe pneumonitis (grade 3–4) is not recommended.

## Rare Toxicities

### Cardiotoxicity

Cardiac irAEs are associated with low incidence, but high mortality rates (Čelutkienė et al., 2020). They could present as pericarditis with pericardial pain, myocarditis with dyspnea due to pulmonary edema or arrhythmias with palpitations, pre-syncope and syncope (Tocchetti et al., 2019; Upadhrasta et al., 2019). Three cases of myocarditis were observed in hemato-oncological patients undergoing ICI therapy. Two were attributed to the combination of pembrolizumab with IMiD and dexamethasone and both of these events were fatal (Martinez-Calle et al., 2018; Mateos M.-V. et al., 2019; Usmani et al., 2019). The third was observed in a phase 1b/2 clinical trial for newly diagnosed (ND) follicular lymphomas and investigated the combination of atezolizumab, obinutuzumab and bendamustine (Younes et al., 2017). In this case, the patient suffered from grade 4 myocarditis and bronchiolitis obliterans and the immediate cause of death was atezolizumab-related cardiac arrest preceded by an episode of dyspnea. (Table 6)

**TABLE 6:** Incidence of rare irAEs in hematological malignancies.

Rare Toxicities						
Author	Condition	Phase	Regimen	Symptom	Incidence	
					all grade	grade ≥ 3
Cardiotoxicity						
Mateos et al. (2019b)	RR MM	3	pembro + pom + dex	myocarditis	1%	1%
Usmani et al. (2019)	ND MM	3	pembro + lena + dex	myocarditis	1%	1%
Younes et al. (2017)	ND FL	1b/2	atezo + obi + benda	myocarditis	3%	3%
Neurotoxicity						
Diefenbach et al. (2020)	RR cHL	1/2	ipili + BV	peripheral sensory neuropathy	69%	4%
			nivo + BV		53%	0%
			ipili + nivo + BV		36%	0%
Cohen et al. (2021)	RR MM	2/3	cetreli + dara	autoimmune encephalitis	11%	0%
D’Souza et al. (2019)	ND MM	2	pembro + lena	neuropathy	7%	0%
Zeidan et al. (2018)	RR MDS	1b	ipili	Bell´s palsy	3%	0%
Other						
Liu et al. (2021)	RR cHL	2	camre + decitabine	myalgia	10%	0%
Shi et al. (2021)	RR PTCL	2	geptanolimab	arthralgia	2%	0%
				AI hemolytic anemia	1%	1%
Diefenbach et al. (2020)	RR cHL	1/2	ipili + nivo + BV	arthritis	5%	5%
Song et al. (2020)	RR cHL	2	tislelizumab	osteoarthritis	1%	NA
				kidney injury	1%	1%
Mei et al. (2020)	RR PMBL	2	camre + GVD	myalgia	4%	0%
Khodadoust et al. (2016)	RR MF, RR SS	2	pembro	arthralgia + arthritis	13%	4%
				corneal ulcer	4%	4%
Zinzani et al. (2019)	RR PMBL	1/2	nivo + BV	kidney injury	7%	3%
Barta et al. (2019)	RR T-NHL	2	pembro	vasculitis	6%	6%
				HLH	6%	6%
Ansell et al. (2019)	RR DLBCL	2	nivo	nephritis + renal dysfunction	NA	4%
Armand et al. (2019)	RR PMBL	1b	pembro	myositis	5%	5%
Davids et al. (2016)	RR hematol. malign.	1/1b	ipili	ITP	4%	0%
Bashey et al. (2009)	cancer post HSCT	1	ipili	polyarthritis	3%	3%
D’Souza et al. (2019)	ND MM	2	pembro + lena	arthralgia	3%	0%
				glomerulonephritis	3%	3%
Davids et al. (2020)	RR hematol. malign.	1	nivo	arthralgia	11%	4%
Daver et al. (2019)	RR AML	2	nivo + azacitidine	HLH	1%	1%
Zeidan et al. (2018)	RR MDS	1b	ipili	myositis	3%	0%

RR, relapsed/refractory; ND, newly diagnosed; cHL, classical Hodgkin lymphoma; FL, follicular lymphoma; PTCL, peripheral T cell lymphoma; NHL- non-Hodgkin lymphoma; MM, multiple myeloma; PMBL, primary mediastinal large B cell lymphoma; DLBCL, diffuse large B cell lymphoma; MF, mycosis fungoides; SS, Sézary syndrome; AML, acute myeloid leukemia; MDS, myelodysplastic syndrome; HSCT, (allogeneic) hematopoietic stem cell transplantation; atezo, atezolizumab; ipili, ipilimumab; nivo, nivolumab; obi, obinutuzumab; benda, bendamustin; BV, brentuximab vedotin; GVD, gemcitabine + vinorelbine + doxorubicin; pembro, pembrolizumab; pom, pomalidomide; lena, lenalidomide; cetreli, cetrelimab; dara, daratumumab; dex, dexamethasone; ida, idarubucin; camre, camrelizumab; AI, autoimmune; HLH, hemophagocytic lymphohistiocytosis; ITP, immune thrombocytopenia.

Diagnostic tools for myocarditis are cardiac biomarkers (troponin and creatinine kinase MB), electrocardiogram, echocardiogram, cardiac MRI and endomyocardial biopsy (Upadhrasta et al., 2019; Pudil et al., 2020). Recently, global longitudinal strain (GLS) has also been considered to be of diagnostic and prognostic value as its decrease may be associated with ICI-related myocarditis (even in cases with preserved left ventricle ejection fraction) and higher probability of major adverse cardiac events (cardiogenic shock, arrest, complete heart block and cardiac death) (Awadalla et al., 2020; Čelutkienė et al., 2020). GLS is derived from transthoracic echocardiogram so no additional examination is required.

There are no definite treatment guidelines for ICI-associated cardiotoxicity. However, close monitoring of the patient and high-dose corticosteroids are essential in the management of these events. Steroid dosage varies from 1 to 2 mg/kg/day of prednisone in non-fulminant myocarditis to 1000mg of intravenous methylprednisolone per day in fulminant cases (Haanen et al., 2017; Upadhrasta et al., 2019). In non-responders, escalation to infliximab, mycophenolate mofetil or anti-thymocyte globulin is recommended. The restart of ICI treatment may be considered only for patients experiencing grade 1 cardiotoxicity (Chhabra and Kennedy, 2021).

### Neurotoxicity

Neurologic irAEs are uncommon, but have many forms. ICI-associated complications involving the peripheral nervous system include polyradiculopathies, neuropathies, myasthenic syndromes and myopathies, whereas the central nervous system can be affected by aseptic meningitis and encephalitis (Chhabra and Kennedy, 2021; Roth et al., 2021). Imaging, cerebrospinal fluid diagnostic, electroencephalography/electromyoneurography and laboratory assessments help exclude tumor progression, vascular complications, infections, epilepsy, metabolic or toxic conditions and side effects of previous or current therapies. Median time to onset of these events ranges from 6 to 13 weeks (Haanen et al., 2017).

Relatively high incidence of mostly grade 1–2 peripheral sensory neuropathy was observed in a phase 1/2 clinical trial investigating the combination of ipilimumab, nivolumab and BV for RR cHL patients (Diefenbach et al., 2020). However, these results should be regarded with caution due to the high neurotoxic potential of BV alone. D´Souza et al. reported a 7% rate of neuropathy in their pembrolizumab-based study (D’Souza et al., 2019). Solitary and non-severe cases of Bell´s palsy and autoimmune encephalitis occurred in blood cancer patients on ICI therapy (Zeidan et al., 2018; Cohen et al., 2021). (Table 6)

If neurological irAEs grade ≥2 are considered, ICI treatment should be interrupted and systemic steroids initiated (Haanen et al., 2017; Chhabra and Kennedy, 2021; Roth et al., 2021). For patients experiencing grade 2 neurotoxicity, it is prednisolone 0.5–1 mg/kg. For greater grades, intravenous (methyl)prednisolone 1–2 mg/kg is recommended. Plasmapheresis or intravenous immunoglobulins (400 mg/kg for 5 days) are indicated for non-responders to steroids with Guillain-Barré syndrome and myasthenia gravis. In the latter case, pyridoxine in an initial dose of 30 mg three times a day is also advised. When the symptoms are not improving or worsening, other immunosuppressants such as azathioprin, cyclosporine, mycophenolate mofetil or even rituximab may be added, but there are no specific guidelines. Since these irAEs are potentially life-threatening, early consultation and cooperation with neurologists is appropriate.

### Rheumatological Toxicity

Arthralgia and myalgia are the most frequently reported rheumatic irAEs, but arthritis, myositis and vasculitis were observed in blood cancer ICI trials as well (Bashey et al., 2009; Khodadoust et al., 2016; Zeidan et al., 2018; Armand et al., 2019; Barta et al., 2019; D’Souza et al., 2019; Davids et al., 2020; Diefenbach et al., 2020; Mei et al., 2020; Song et al., 2020; Liu et al., 2021; Shi et al., 2021). (Table 6) These adverse events are more likely to occur in ICIs acting on the PD-1 axis and may have later onset (Chhabra and Kennedy, 2021). Mild to moderate cases may be managed with analgesics or low-dose prednisolone (10–20 mg), more severe cases require cooperation with rheumatologists and higher doses of oral or intravenous corticosteroids or addition of other immunosuppressive agents such as infliximab, azathioprin, mycophenolate mofetil etc (Haanen et al., 2017; Jamal et al., 2020).

### Nephrotoxicity

Rare irAEs also include renal impairments. The most common presentation is acute kidney injury (AKI) due to acute tubulointerstitial nephritis (Tinawi and Bastani, 2020; Chhabra and Kennedy, 2021). The mechanism is still unclear, but three risk factors were observed: 1) concomitant use of proton pump inhibitors, 2) low baseline estimated glomerular filtration rate (<30 ml/min/1.73 m^2^), 3) dual immune checkpoint blockade. Laboratory findings suggesting ICI-associated AKI could be microhematuria, subnephrotic range proteinuria, sterile pyuria and eosinophilia. Generally, median onset of AKI for anti-CTLA-4 mAbs is 2 months and for PD-1 axis agents, 3–10 months. In our review of ICI blood cancer clinical trials, treatment-related AKI/nephritis occurred only in patients receiving anti-PD-1 treatment and one case was fatal (Ansell et al., 2019; Armand et al., 2019; Daver et al., 2019; D’Souza et al., 2019; Song et al., 2020). (Table 6)

ICI therapy should be withheld in grade ≥2 AKI (creatinine >1.5x baseline/ULN) and may be restarted after restitution to grade 1, but more severe cases must be dealt with caution (Haanen et al., 2017; Tinawi and Bastani, 2020). Oral prednisolone 0.5–1 mg/kg should be initiated in grade 2 AKI (creatinine 1.5-3x baseline/ULN) and intravenous (methyl)prednisolone 1–2 mg/kg in grade ≥3 AKI (creatinine >3x baseline/ULN). Kidney biopsy is not vital for determining the diagnosis, but may help to avoid unnecessary steroid treatment and ICI therapy discontinuation in dubious cases. Consultation with nephrologists would be appropriate.

### Other Toxicities

Solitary cases of other types of irAEs were described in ICI blood cancer clinical trials. One patient with RR mycosis fungoides/Sézary syndrome who was receiving pembrolizumab experienced grade 3 corneal ulcer (Khodadoust et al., 2016). Incidents of autoimmune hematologic toxicity included newly diagnosed immune thrombocytopenia during ipilimumab therapy, autoimmune hemolytic anemia grade 4 during geptanolimab therapy (anti-PD-1 mAb) and one grade 3 and one lethal case of hemophagocytic lymphohistiocytosis in clinical studies investigating pembrolizumab monotherapy in T cell lymphomas and the combination of nivolumab with azacitidine for acute myeloid leukemia patients, respectively (Davids et al., 2016; Barta et al., 2019; Daver et al., 2019; Shi et al., 2021). (Table 6)

## Conclusion

Immune checkpoint blockade completely revolutionized the landscape of solid tumor oncology treatment. However, its journey in hemato-oncology has been much slower and more difficult. The initial great success in cHL and later PMBL, which led to accelerated FDA approval of nivolumab and pembrolizumab in these indications, was overshadowed by the general hold that was placed on large clinical trials investigating the promising combination of ICIs and IMiDs in the multiple myeloma setting due to suspicious treatment-related toxicity. Moreover, dual immune checkpoint blockade did not fulfil expectations and did not show increased benefit for patients suffering from hematological malignancies, only greater toxicity was reported. Aside from cHL and PMBL, ICI monotherapy did not prove to be effective either. Nevertheless, the future of ICIs in hemato-oncology seems to lie in combinations, either with conventional chemotherapy or other targeted agents such as mAbs, CAR T cells, PI3κ inhibitors, antibody-drug conjugates or bispecific antibodies as many ongoing studies and their promising efficacy results demonstrate. Therefore, hemato-oncologists should be prepared to work with ICIs more often and learn to recognize, diagnose and manage their specific irAEs.

Generally, the incidence and distribution of irAEs is approximately similar between solid tumor oncology and hemato-oncology. However, the exact rates may differ due to smaller amounts of treated patients and the fact that most of the safety data came from phase 1 and 2 clinical trials. The only available phase 3 safety data come from the controversial KEYNOTE-183 and KEYNOTE-185 studies (Mateos M.-V. et al., 2019; Usmani et al., 2019). The first one investigated the benefit of adding pembrolizumab to pomalidomide and dexamethasone in RR MM patients while the second one was for ND MM and studied lenalidomide and dexamethasone with or without pembrolizumab. These combinations proved toxic, when both clinical trials were suspended prematurely because of higher treatment-related mortality rates in experimental arms. Even if no specific adverse event of the ICI and IMiD combination has been unveiled to date, it is worth mentioning that the only two cases of grade 5 ICI-related myocarditis reported from prospective ICI blood cancer trials came from these two (1 from each). Investigation of another combination, daratumumab (anti-CD38 mAb) with PD-1 or PD-L1 inhibitors, was abandoned based on the unsatisfying results from a phase 1b/2 LUC2001 study of atezolizumab with or without daratumumab for advanced or metastatic non-small-cell lung cancer (NCT03023423) (Cohen et al., 2021). The cohort which received both agents did not reach superior outcome and there was also an imbalance of deaths in the experimental arm even if they weren´t considered treatment-related. Although this study was targeting solid tumors, the decision of the investor affected all similar ongoing studies, including hemato-oncological (mainly MM again).

Agents acting on PD-1 axis are used in hemato-oncology more often than CTLA-4 inhibitors, so irAEs such as hyper/hypothyroidism or pneumonitis may occur more frequently. Nevertheless, blood cancer specialists should be aware of all possible types of irAEs. Mostly, it is a diagnosis per exclusionem, so the physician is required to eliminate all other feasible causes. The majority of irAEs are mild to moderate, can be managed symptomatically or by oral steroids and mainly do not lead to permanent ICI discontinuation. On the other hand, fatal and potentially lethal irAEs were also reported, especially myocarditis, pneumonitis, neurologic events, hypophysitis, acute kidney injury and Stevens-Johnson syndrome. These events usually require permanent cessation of ICI treatment, rapid administration of high dose corticosteroids or other immunosuppressants (infliximab, mycophenolate mofetil, tacrloimus etc.) and multidisciplinary approach.
